# Additive Benefit of Guideline-Directed Medical Therapies at Discharge in Reducing 30-Day Readmissions in Heart Failure

**DOI:** 10.1016/j.jacadv.2025.102411

**Published:** 2025-12-04

**Authors:** Andrew Willeford, Barry Greenberg, Zaid Yousif

**Affiliations:** a UC San Diego Skaggs School of Pharmacy and Pharmaceutical Sciences, La Jolla, California, USA; b UC San Diego Health, Department of Cardiology, San Diego, California, USA

**Keywords:** guideline-directed medical therapy, heart failure, hospital readmission, patient discharge, registries

## Abstract

**Background:**

Hospitalization-related costs for heart failure (HF) are a major contributor to the overall health care expenditure in the United States. Despite recommendations, guideline-directed medical therapy (GDMT) is underutilized at discharge in eligible patients, likely contributing to high readmission rates. Describing the effect of GDMT use at discharge may better inform institutions on the value of implementing focused therapy initiatives.

**Objectives:**

This study aimed to examine the association between the number of active GDMT prescriptions at discharge and 30-day readmissions.

**Methods:**

This retrospective cohort study analyzed 2,121 index hospitalizations for HF across 5 institutions from February 1, 2021, to October 31, 2024. Patients included were adults with a baseline left ventricular ejection fraction ≤40%. The primary outcome was 30-day all-cause readmission, and the secondary outcome was 30-day HF readmission. Multivariable mixed-effects Cox proportional hazard models were used to assess the association between GDMT count and readmission rates.

**Results:**

Increased GDMT count at discharge was associated with significantly lower hazard of 30-day all-cause readmission: 1 vs 2 GDMT (HR: 0.79; 95% CI: 0.64-0.97), 1 vs 3 GDMT (HR: 0.70; 95% CI: 0.55-0.90), and 1 vs 4 GDMT (HR: 0.56; 95% CI: 0.40-0.77). Trends were similar for HF-specific readmissions.

**Conclusions:**

Increasing number of GDMT classes prescribed at discharge was associated with a gradient reduction in 30-day all-cause and HF readmissions, with a stronger effect seen with higher GDMT counts. Implementing comprehensive GDMT strategies at discharge may reduce health care costs and enhance institutional performance under national quality metrics.

Approximately 6.7 million adults in the United States are currently living with heart failure (HF), a number projected to increase to 8.5 million by 2030.^1^ Total costs for caring for patients with HF are expected to reach $70 billion by 2030, with the majority of costs attributable to hospitalization.^2^ Patients hospitalized for HF are likely to be readmitted with studies showing all-cause 30-day readmission rates as high as 25%.^3^ These findings emphasize a need for inpatient pathways to reduce HF readmissions and provide cost-effective care.

Guideline-directed medical therapy (GDMT) for HF with reduced ejection fraction (HFrEF) includes evidence-based beta-blockers (BB), mineralocorticoid receptor antagonists (MRA), sodium glucose co-transporter 2 inhibitors (SGLT2i), and inhibitors of the renin-angiotensin system which include angiotensin-converting enzyme inhibitors (ACEI), angiotensin receptor blockers (ARB), and angiotensin receptor-neprilysin inhibitors (ARNI).^4^ While the effect of the individual classes of GDMT appears to be additive in reducing 90-day HF readmissions, it remains unclear whether a similar effect is observed at 30 days.^5^ Despite guideline recommendations to initiate therapy in hospitalized patients and evidence from multiple clinical trials demonstrating the safety and efficacy of inpatient initiation of GDMT,^4^ real-world studies have shown that it is underutilized in eligible patients being discharged from the hospital.^5^, ^6^, ^7^ This gap between clinical recommendations and real-world utilization highlights the importance of describing the impact of inpatient initiation and optimization of comprehensive GDMT, a modifiable practice that may help to prevent 30-day readmissions.

The 30-day readmission rate is a major metric for the Centers for Medicare and Medicaid Services in grading an institution’s quality of care and determining reimbursement.^8^^,^^9^ Understanding the benefits of GDMT use at discharge may better inform institutions about the value in implementing inpatient initiatives aimed at reducing 30-day readmissions in patients with HF. Herein this study, we utilize the UC Health Data Warehouse (UCHDW) to analyze active GDMT prescriptions at discharge and assess their association with 30-day all-cause and 30-day HF readmission rates.^10^

## Methods

### Data source

This retrospective cohort study utilized the UCHDW. The UCHDW holds data on over 9 million patients seen at a University of California facility since 2012. It includes 375 million encounters, over 1 billion procedures, more than 1.3 billion medications prescribed, dispensed, and administered, more than 5.2 billion vital signs and laboratory test results, and over 1.1 billion diagnosis codes. These data are stored in the Observational Medical Outcomes Partnership data model. Known identifiers from patient data used in the present study have been removed in compliance with the Health Insurance Portability and Accountability Act. The warehouse is managed under the oversight of Institutional Review Boards and information security teams at the University of California campuses. Researchers do not need to obtain Institutional Review Board approval as the data have been determined to be not human subjects research under the Common Rule.

### Study cohort

We identified index hospital stays throughout the University of California Healthcare facilities from February 1, 2021, to October 31, 2024, with a principal admission diagnosis of HF. Diagnoses of HF were identified using the International Classification of Diseases-10th Revision-Clinical Modification (ICD-10-CM) codes I11.0∗, I13.0∗, I13.2∗, and I50∗. The start date for the study period was chosen based on the date that SGLT2is were deemed GDMT.^11^ Included patients were ≥18 years of age with a baseline left ventricular ejection fraction (LVEF) ≤40% as determined by echocardiography conducted within 1 year before index hospitalization. Patients were excluded if they met any of the following criteria: death during hospitalization, absence of GDMT at discharge, no follow-up within the health system postdischarge within 60 days, discharge to hospice care or with intravenous inotropes, or a history of cardiac amyloidosis, hypertrophic cardiomyopathy, end-stage renal disease, heart transplant, or ventricular assist device. Exclusion of patients not discharged on GDMT was intended to reduce the risk for residual confounding. Patient-level demographics and comorbidities were assessed on the day of discharge for each index hospitalization. ICD-10-CM codes were used to identify selected comorbidities as described in Supplemental Table 1. Baseline health and general comorbidities burden were evaluated using the Elixhauser Comorbidity Index, which incorporates 30 disease states to create a score ranging from 0 to 30, with higher scores indicating a higher risk for adverse outcomes.^12^^,^^13^ The last recorded vital signs and laboratory values from each hospitalization were also extracted. In this analysis, a prescription was considered active if it met the following criteria: 1) it was intended for outpatient dispensing; 2) it was prescribed before or during the index hospitalization; and 3) its end date was beyond the hospital discharge date.

### Outcomes

The primary outcome was the association of GDMT count (ie, 1, 2, 3, or 4 classes) on 30-day all-cause readmissions. The secondary outcome was the association of GDMT count on 30-day readmissions with HF as the primary diagnosis. To examine the impact of survivorship bias, we conducted an exploratory analysis by examining the following composite outcomes: 1) all-cause death or 30-day all-cause readmission; and 2) all-cause death or 30-day readmission with HF as the primary diagnosis. For each index hospitalization, we counted the first unplanned readmission within 30 days from the date of discharge. Death was also assessed within 30 days of hospital discharge. The 4 GDMT classes were defined as: 1) BB; 2) ACEI/ARB/ARNI; 3) MRA; and 4) SGLT2i in accordance with current practice guidelines.^4^ All readmissions were confirmed to be unplanned by reviewing the primary ICD-10-CM diagnosis codes for each hospital admission and verifying that they were not indicative of elective procedures or hospitalization.

### Statistical analyses

Patient-level characteristics for each index hospitalization were combined and calculated as frequency and percentage for categorical variables, mean ± SD for normally distributed continuous variables, and median and IQR for non-normally distributed variables. Normality was assessed using Q–Q plots or by considering the sample size. Continuous variables with 2 levels were assessed using independent two-sample *t*-test or Wilcoxon signed rank test, as appropriate. Continuous variables with more than 2 levels were assessed using 1-way analysis of variance or Kruskal-Wallis test, as appropriate. Categorical variables with all expected cell counts ≥5 were analyzed using chi-square test. Categorical variables with all expected cell counts <5 were analyzed using Fisher exact test. Missing values were imputed using the median of the corresponding GDMT group. Mixed-effects Cox proportional hazards models were used to estimate the association between GDMT count and the primary, secondary, and exploratory outcomes. Mixed-effects models account for data clustering by incorporating random effects to model correlations within grouped data.^14^ In this study, we incorporated random effects to account for clustering at both the patient and hospital system levels. HRs and 95% CI were used to quantify the strength and direction of the association. Forest plots and Kaplan-Meier curves were used to illustrate the association of GDMT count on outcome risk. In the primary and secondary outcomes, patients were censored at the time of death. We took a rigorous scientific approach to account for potential confounding variables and the general health status of patients by incorporating the following variables in all models based on literature review, clinical judgment, and availability in the data source: race, age, sex, prior hospitalization for HF, length of hospital stay, LVEF, systolic blood pressure, estimated glomerular filtration rate, heart rate, body mass index, potassium, sodium, atrial fibrillation, coronary artery disease, chronic obstructive pulmonary disease, diabetes type 2, hypertension, ischemic heart disease, stroke, implantable cardioverter defibrillator, Elixhauser Comorbidity Index, and health insurance.^15^ All statistical tests were 2-tailed, and statistical significance was defined as an alpha <0.05. All analyses were performed using R statistical software version 4.3.2. Mixed-effects Cox proportional hazard models were fitted using *coxme* 2.2-22 package. Proportional hazards assumption was tested using the implementation of Grambsch et al^16^ in the *survival* 3.8-3 package.

## Results

### Cohort characteristics

**Figure 1:**
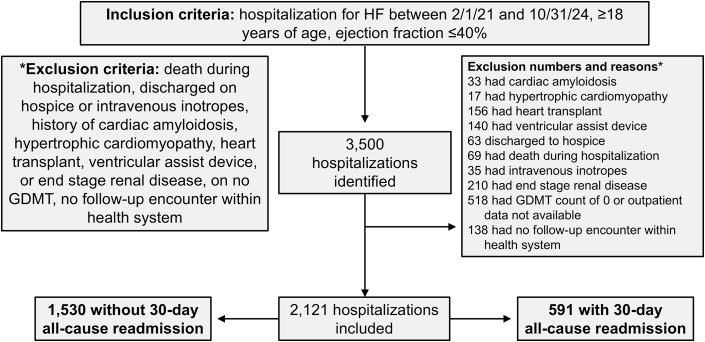
**Study Flow Diagram** GDMT = guideline-directed medical therapy; HF = heart failure.

A total of 3,500 index hospitalizations were identified, of which 2,121 hospitalizations among 1,152 unique patients were included in the final analysis (Figure 1). Among the included hospitalizations, 27.9% had a 30-day all-cause readmission and 19.8% had a 30-day HF readmission (Central Illustration). Patient characteristics are presented in Table 1 and organized by GDMT count (ie, monotherapy, dual therapy, triple therapy, and quad therapy). Overall, the patients were a mean 59.3 ± 14.5 years of age, 25.6% female, and 41.9% White. Significant differences among the 4 groups were observed in age, body mass index, LVEF, systolic blood pressure, potassium, estimated glomerular filtration rate, hemoglobin, Elixhauser Comorbidity Index, presence of atrial fibrillation, type 2 diabetes, and health insurance. The distributions of ACEI/ARB, ARNI, BB, MRA, SGLT2i, and loop diuretics among the 4 groups were also significantly different. Characteristics for patients who did not have follow-up within 60 days are shown in Supplemental Table 2.

**Central Illustration:**
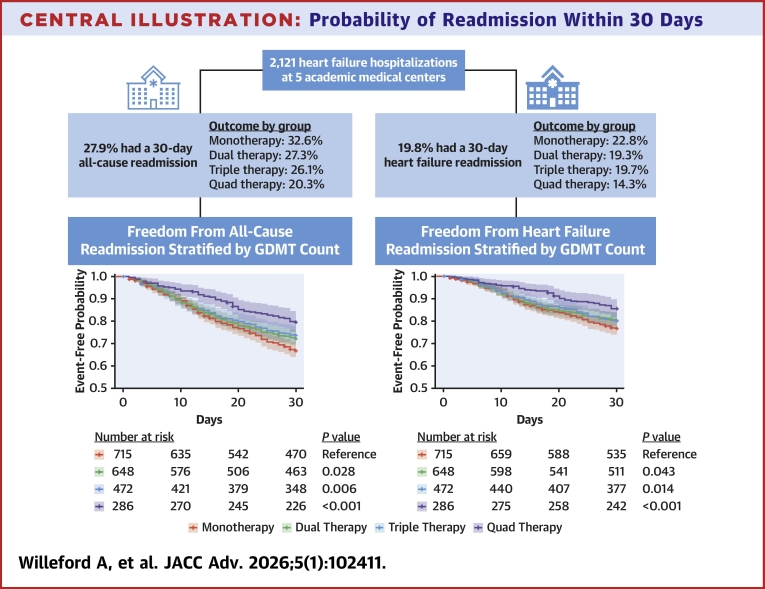
**Probability of Readmission Within 30 Days** A total of 2,121 index hospitalizations for heart failure among 5 large academic medical centers was identified, with 27.9% having a 30-day all-cause readmission and 19.8% having a 30-day heart failure readmission. Kaplan-Meier curves depict the event-free probability with patients being censored at the time of death as indicated by crosses. Increasing number of GDMT classes prescribed at discharge was associated with a gradient reduction in 30-day readmissions, with a stronger effect seen with higher GDMT counts. Adjusted *P* values for each GDMT group compared to monotherapy are shown. Abbreviation as in Figure 1.

**Table 1:** Baseline Characteristics

	Monotherapy (n = 715)	Dual Therapy (n = 648)	Triple Therapy (n = 472)	Quad Therapy (n = 286)	*P* Value
Age, y (SD)	62.3 (14.3)	60.1 (14.4)	56.3 (14.4)	54.9 (13.2)	<0.001
Female, n (%)	199 (27.8)	161 (24.8)	118 (25.0)	65 (22.7)	0.300
Body mass index, kg/m^2^ (SD)	28.1 (7.7)	28.8 (7.7)	29.2 (8.3)	30.2 (9.1)	0.005
Ejection fraction, % (SD)	26.5 (8.4)	24.3 (8.2)	23.8 (7.8)	23.2 (7.6)	<0.001
Systolic blood pressure, mm Hg (SD)	114 (19)	112 (18)	110 (17)	107 (16)	<0.001
Heart rate, beats/min (SD)	81 (16)	83 (14)	82 (15)	82 (17)	0.200
Sodium, mEq/L (SD)	136 (4)	136 (4)	136 (3)	136 (3)	0.028
Potassium, mEq/L (SD)	4.1 (0.5)	4.1 (0.5)	4.2 (0.4)	4.2 (0.4)	0.049
Preadmission HFH, count (SD)	2.6 (3.4)	2.7 (3.1)	2.8 (2.9)	2.8 (2.6)	0.110
Estimated GFR, mL/min/1.73^2^ (SD)	50.2 (20.1)	56.5 (18.9)	63.0 (17.4)	64.6 (18.4)	<0.001
Hemoglobin, g/dL (SD)	12.0 (2.3)	12.5 (2.3)	13.0 (2.2)	13.7 (2.4)	<0.001
Length of stay, days (SD)	7.1 (6.5)	6.9 (6.1)	6.8 (7.4)	6.6 (6.4)	>0.900
Elixhauser Comorbidity Index, score (SD)	7.4 (2.4)	7.1 (2.4)	6.9 (2.3)	7.0 (2.5)	0.006
Race, n (%)					
White	321 (44.9)	266 (41.0)	205 (43.4)	96 (33.6)	0.145
Black	203 (28.4)	203 (31.3)	135 (28.6)	92 (32.2)	
Other	111 (15.5)	106 (16.4)	82 (17.4)	64 (22.4)	
Asian	60 (8.4)	46 (7.1)	34 (7.2)	20 (7.0)	
Unknown	11 (1.5)	13 (2.0)	7 (1.5)	6 (2.1)	
Native Hawaiian or Pacific Islander	6 (0.8)	7 (1.1)	4 (0.8)	7 (2.4)	
Native American	3 (0.4)	7 (1.1)	5 (1.1)	1 (0.3)	
Medical history, n (%)					
Atrial fibrillation/flutter	343 (48.0)	314 (48.5)	195 (41.3)	120 (42.0)	0.033
Coronary artery disease	371 (51.9)	321 (49.5)	219 (46.4)	134 (46.9)	0.200
Chronic obstructive pulmonary disease	151 (21.1)	123 (19.0)	73 (15.5)	59 (20.6)	0.100
Type 2 diabetes	96 (13.4)	102 (15.7)	86 (18.2)	66 (23.1)	0.002
Hypertension	110 (15.4)	101 (15.6)	63 (13.3)	35 (12.2)	0.400
Stroke	17 (2.4)	24 (3.7)	13 (2.8)	4 (1.4)	0.200
Ischemic heart disease	412 (57.6)	353 (54.5)	242 (51.3)	149 (52.1)	0.140
Implantable cardioverter defibrillator	213 (29.8)	203 (31.3)	141 (29.9)	65 (22.7)	0.060
Insurance, n (%)					
Medicaid	307 (42.9)	309 (47.7)	275 (58.3)	180 (62.9)	<0.001
Multiple	247 (34.5)	218 (33.6)	114 (24.1)	62 (21.7)	
Medicare Advantage	56 (7.9)	42 (6.5)	40 (8.6)	23 (8.1)	
Commercial	53 (7.4)	55 (8.5)	25 (5.4)	12 (4.2)	
Medicare	44 (6.2)	20 (3.1)	12 (2.6)	6 (2.1)	
Veterans Affairs	5 (0.7)	1 (0.2)	1 (0.2)	2 (0.7)	
Unknown	3 (0.4)	3 (0.5)	5 (1.1)	1 (0.3)	
Medications, n (%)					
ACEI/ARB	76 (10.6)	200 (30.9)	201 (42.6)	94 (32.9)	<0.001
ARNI	78 (10.9)	179 (27.6)	201 (42.6)	192 (67.1)	<0.001
BB	382 (53.4)	437 (67.4)	388 (82.2)	286 (100)	<0.001
MRA	100 (14.0)	228 (35.2)	357 (76.6)	286 (100)	<0.001
SGLT2i	79 (11.0)	252 (38.9)	269 (57.0)	286 (100)	<0.001
Loop diuretic	536 (75.0)	512 (79.0)	395 (83.6)	261 (91.3)	<0.001

The Elixhauser Comorbidity Index is a measure of overall comorbidity burden using ICD-10 codes for 30 disease states. Scores range from 0 to 30, with higher scores indicating a higher risk for adverse outcomes.

ACEI = angiotensin-converting enzyme inhibitor; ARB = angiotensin receptor blocker; ARNI = angiotensin receptor-neprilysin inhibitor; BB = beta-blockers; GFR = glomerular filtration rate; HFH = heart failure hospitalization; ICD-10 = International Classification of Diseases-10th Revision; MRA = mineralocorticoid receptor antagonists; SGLT2i = sodium glucose co-transporter 2 inhibitors.

### Association of GDMT count on 30-day all-cause or HF readmissions

**Figure 2:**
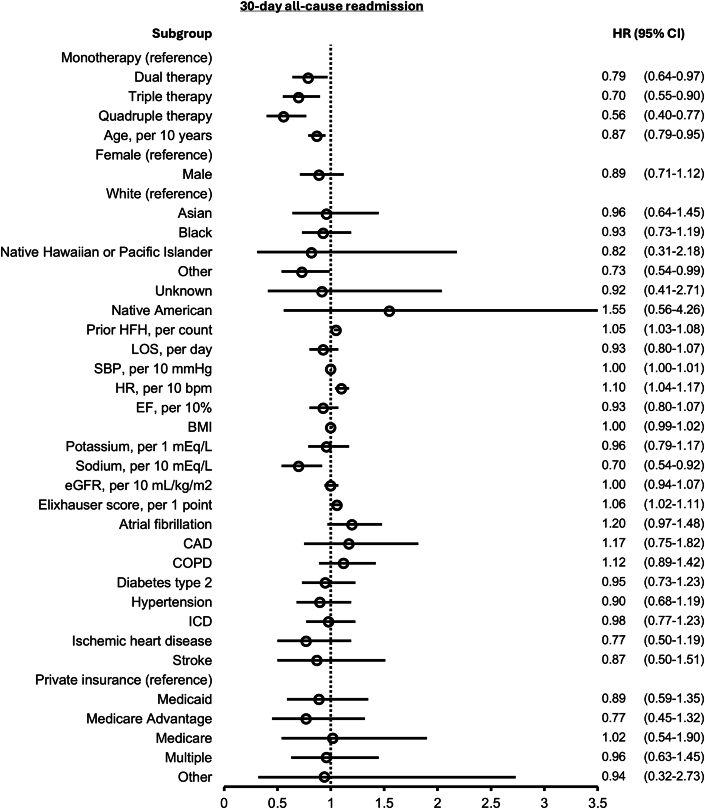
**Forest Plot for 30-Day All-Cause Readmission** HRs and 95% CIs are shown. BMI = body mass index; CAD = coronary artery disease; COPD = chronic obstructive pulmonary disease; EF = ejection fraction; eGFR = estimated glomerular filtration rate; HFH = prior hospitalization for heart failure; HR = heart rate; ICD = implantable cardioverter-defibrillator; LOS = length of hospital stay; SBP = systolic blood pressure.

The probability of 30-day all-cause readmission by GDMT count is depicted in the Central Illustration. Patients with multiple GDMT were associated with lower hazards of 30-day all-cause readmission compared to those with just 1 GDMT. After adjusting for covariates, patients discharged with 2 GDMT demonstrated reduced hazards of readmission (HR: 0.79; 95% CI: 0.64-0.97), with further reductions observed in discharges with 3 GDMT (HR: 0.70; 95% CI: 0.55-0.90) and 4 GDMT (HR: 0.56; 95% CI: 0.40-0.77) (Figure 2). Comparable results were observed when assessing the composite endpoint of death or 30-day all-cause readmission (Supplemental Figure 1). Crude analyses of both outcomes are presented in Supplemental Tables 3 and 4.

**Figure 3:**
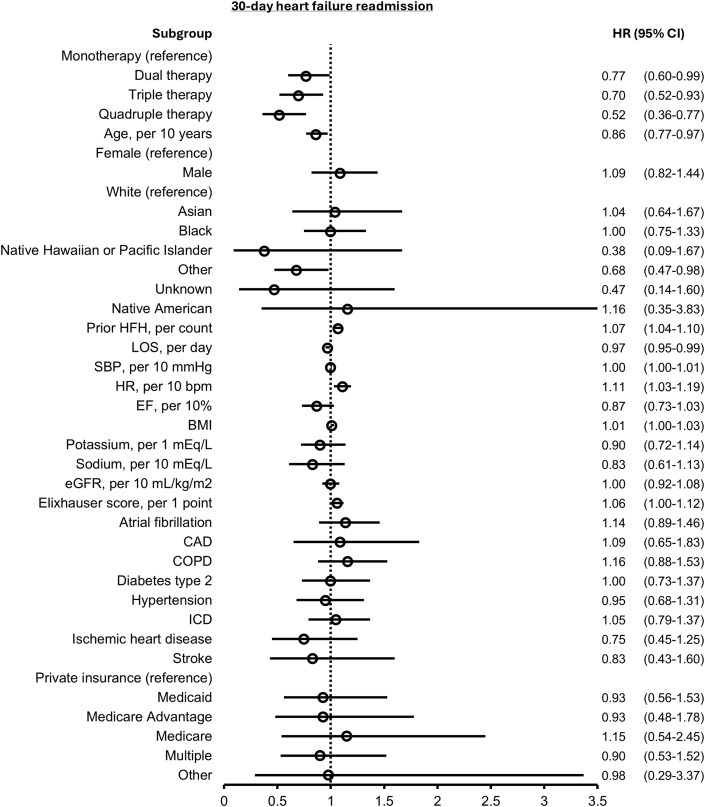
**Forest Plot for 30-Day Heart Failure Readmission** HRs and 95% CIs are shown. Abbreviations as in Figure 2.

The probability of 30-day HF readmission is depicted in the Central Illustration. After adjusting for covariates, patients on 2 GDMT (HR: 0.77; 95% CI: 0.60-0.99), 3 GDMT (HR: 0.70; 95% CI: 0.52-0.93), or 4 GDMT (HR: 0.52; 95% CI: 0.36-0.77) had significantly lower hazards of HF readmission compared to 1 GDMT (Figure 3). Comparable results were observed when assessing the composite endpoint of death or 30-day HF readmission (Supplemental Figure 2). Crude analyses of outcomes related to HF readmission and death or HF readmission are shown in Supplemental Tables 5 and 6.

## Discussion

The gap between clinical recommendations and underutilization of GDMT in real-world patients with HF being discharged from the hospital highlights the importance of describing the impact of inpatient therapy initiation on outcomes meaningful to institutions. In this analysis of 2,121 index hospitalizations for HF across 5 large academic medical centers, the use of an increasing number of classes of GDMT was associated with a gradient reduction in the hazards of 30-day all-cause readmission and 30-day HF readmission. This finding highlights the importance of optimizing GDMT in the hospital to enhance patient outcomes and potentially lower health care system costs.

HF significantly increases the risk of developing or worsening other diseases given patients with HF often have comorbidities that can lead to complications in other organ systems.^17^, ^18^, ^19^ Furthermore, treating HF with GDMT has been linked to lowering comorbidity burden which may explain the observed effect of more GDMT leading to fewer all-cause 30-day readmissions in this study.^20^ Another explanation for our observation relates to our methodology of using ICD-10 codes to identify readmission diagnoses. Research has suggested that hospital coding practices may obscure HF as the primary readmission diagnosis.^21^ This means that patients admitted for reasons other than HF might still have prior HF as the underlying cause driving their readmission. Consequently, the observed effect, where more GDMT appears to reduce 30-day all-cause readmissions, may stem from treating underlying HF that is inaccurately coded at the time of admission.

The association of more GDMT on fewer 30-day readmissions has important implications on patient morbidity in addition to financial impact on health systems. The Centers for Medicare and Medicaid Services uses readmission rates as a proxy for quality of care and financially penalizes hospitals with high 30-day readmission rates under the Hospital Readmissions Reduction Program.^8^^,^^9^ Our data demonstrated that more classes of GDMT at discharge were associated with further lower hazard for 30-day readmission, suggesting that patients discharged on optimal evidence-based therapies are likely to perform better on national benchmarks. It further highlights that implementation of full GDMT could translate into fewer readmissions, minimizing hospital costs tied to repeat hospitalizations.

The study findings lend support to initiatives focused on addressing the underuse of GDMT in patients with HF, ultimately helping to reduce health care system costs. Research has shown that initiating GDMT during hospitalization is safe and effective,^17^^,^^18^ and achieving comprehensive therapy is feasible, as demonstrated by several innovative models.^22^, ^23^, ^24^ Proper postdischarge transitions of care should also be emphasized although published strategies have shown both positive and neutral results.^25^, ^26^, ^27^, ^28^, ^29^ This has led to suggestions of the need for a combined focus on both in-hospital and clinic-based initiation of quadruple therapy.^30^ Our finding that 30-day HF readmission rates decreased stepwise with increasing GDMT count underscores the cumulative benefits of comprehensive GDMT in managing HF.^31^

### Study limitations

This study has several important limitations. First, we were unable to calculate risk-standardized readmission rates in the same manner as those used in the Hospital Readmissions Reduction Program, which may limit the comparability of our findings with national benchmarks.^32^ Second, while this study is sourced from 5 large academic medical centers, findings may not extend to all centers in the United States given the study institutions are localized to the West Coast. Additionally, readmissions to outside hospitals could not be captured, potentially underestimating the study event rates. Third, we were unable to account for changes in GDMT postdischarge, which may impact the outcomes related to 30-day readmissions. Patients may have had alterations in their GDMT regimen following hospital discharge due to clinical events, follow-up care, or medication intolerance, which were not capturable in our registry analysis. Furthermore, the registry does not capture the type of postdischarge care received, such as whether patients were followed by cardiology specialists, transition of care clinics, internal medicine providers, or other care settings. Variations in the quality and type of follow-up care may have influenced the rates of 30-day readmissions. Additionally, complete prescription dispensing data are not available in the registry; thus, we were unable to assess patient adherence to prescribed GDMT after discharge through measures such as the proportion of days covered.^33^ Medication nonadherence is a known factor affecting the effectiveness of GDMT, and our inability to measure this may have introduced bias in evaluating the true benefit of GDMT on readmissions.^34^ Similarly, we could not identify and include only patients eligible for all 4 GDMT classes. However, we incorporated the variables used to assess eligibility (eg, potassium, renal function, blood pressure, and heart rate) in our multivariable analysis to account for potential confounding. Lastly, death serves as a competing risk in our analysis and could result in overestimation of GDMT impact on the hazard of readmission. However, the exploratory outcomes, which were a composite of death or readmission, showed comparable results to the primary and secondary outcomes. This suggests that the observed associations are likely robust and not solely driven by survivorship bias.

## Conclusions

Our analysis of 2,121 index hospitalizations for HF demonstrates that increasing the use of GDMT at discharge is associated with a gradient reduction in 30-day all-cause and HF-related readmissions. This finding emphasizes the critical role of comprehensive GDMT in optimizing patient outcomes and highlights an opportunity to improve quality of care and reduce financial penalties under programs such as the Hospital Readmissions Reduction Program. By leveraging the inpatient setting for initiation and optimization of GDMT, health systems may effectively address gaps in therapy utilization, reduce readmissions, and minimize health care costs. Future efforts should focus on integrated strategies, combining in-hospital interventions with robust postdischarge support to maximize the benefits of GDMT and improve the overall care continuum for patients with HF.

Perspectives **COMPETENCY IN PATIENT CARE AND SYSTEMS-BASED PRACTICE:** The findings of this study support the importance of optimizing GDMT prior to hospital discharge in patients with HFrEF. Increasing the number of GDMT classes prescribed at discharge was associated with a stepwise reduction in 30-day all-cause and HF readmissions, key quality metrics tied to both patient outcomes and institutional reimbursement. These results reinforce that inpatient initiation and optimization of GDMT not only improve short-term clinical outcomes but also represent a modifiable evidence-based strategy to enhance the quality and value of care for patients with HFrEF.

**TRANSLATIONAL OUTLOOK:** Implementing structured inpatient pathways to ensure appropriate use of GDMT may reduce preventable readmissions and contribute to better transitions of care.

## Funding support and author disclosures

The authors have reported that they have no relationships relevant to the contents of this paper to disclose.
