# Supplementary material for: Additive Benefit of Guideline-Directed Medical Therapies at Discharge in Reducing 30-Day Readmissions in Heart Failure
Source: JACC Adv. 2025 Dec 4;5(1):102411. doi: 10.1016/j.jacadv.2025.102411 (PMC12721035; doi:10.1016/j.jacadv.2025.102411)
Supplement: Supplemental Tables 1-6 and Supplemental Figures 1 and 2 [file mmc1.docx]

| Supplemental Table 1. ICD-10 Codes Used to Identify Diagnoses | |
| --- | --- |
| Diagnoses | **ICD-10 Code** |
| Atrial fibrillation/flutter | I48* |
| Cardiac amyloidosis | E85.8* |
| Chronic obstructive pulmonary disease | J44* |
| Coronary artery disease | I25* |
| End-stage renal disease | N18.6 |
| Heart transplant | Z94.1 |
| Hypertension | I10, I11.0, I11.9, I12.0, I12.9 |
| Hypertrophic cardiomyopathy | I42.1, I42.2 |
| Implantable cardioverter defibrillator | Z95.810 |
| Ischemic heart disease | I20*, I21*, I22*, I23*, I24* |
| Stroke | I63*, I66* |
| Type 2 diabetes | E11* |
| Ventricular assist device | Z95.811 |

| Supplemental Table 2. Baseline Characteristics of Patients Excluded for No Follow-Up | |
| --- | --- |
| Characteristic | **n = 138** |
| Age, years (SD) | 56.8 (13.8) |
| Female, n (%) | 35 (25.3) |
| Body-mass index, kg/m^2^ (SD) | 29.1 (8.2) |
| Ejection fraction, % (SD) | 24.7 (8.1) |
| Systolic blood pressure, mmHg (SD) | 111 (18) |
| Heart rate, beats per minute (SD) | 84 (15) |
| Sodium, mEq/L (SD) | 137 (3) |
| Potassium, mEq/L (SD) | 4.2 (0.5) |
| Pre-admission HFH, count (SD) | 1.8 (1.7) |
| Estimated GFR, mL/min/1.73^2^ (SD) | 61.4 (20.9) |
| Hemoglobin, g/dL (SD) | 13.0 (2.2) |
| Length of stay, days (SD) | 5.6 (5.4) |
| Elixhauser Comorbidity Index, score (SD) | 6.4 (2.2) |
| Race, n (%) | |
| White | 63 (45.7) |
| Black | 41 (29.7) |
| Other | 22 (15.9) |
| Asian | 8 (5.8) |
| Native Hawaiian or Pacific Islander | 2 (1.4) |
| Native American | 2 (1.4) |
| Medical history, n (%) | |
| Atrial fibrillation/flutter | 46 (33.3) |
| Coronary artery disease | 53 (38.4) |
| Chronic obstructive pulmonary disease | 37 (26.8) |
| Type 2 diabetes | 23 (16.7) |
| Hypertension | 10 (7.2) |
| Stroke | 3 (2.2) |
| Ischemic heart disease | 57 (41.3) |
| Implantable cardioverter defibrillator | 16 (11.6) |
| Insurance, n (%) | |
| Medicaid | 88 (63.8) |
| Multiple | 32 (23.2) |
| Medicare Advantage | 9 (6.5) |
| Commercial | 5 (3.6) |
| Medicare | 4 (2.9) |
| Medications, n (%) | |
| ACEi/ARB | 50 (36.2) |
| ARNI | 37 (26.8) |
| BB | 101 (73.2) |
| MRA | 57 (41.3) |
| SGLT2i | 52 (37.7) |
| Loop diuretic | 110 (79.7) |
| Values are mean, standard deviation, numbers, and percentages. The Elixhauser Comorbidity Index is a measure of overall comorbidity burden using ICD-10 codes for 30 disease states. Scores range from 0 to 30, with higher scores indicating a higher risk for adverse outcomes. Abbreviations: ACEi = angiotensin-converting enzyme inhibitor; ARB = angiotensin receptor blocker; ARNI = angiotensin receptor-neprilysin inhibitor; BB = beta blocker; GDMT = guideline-directed medical therapy; GFR = glomerular filtration rate; HFH = heart failure hospitalization; MRA = mineralocorticoid receptor antagonist; SD = standard deviation; SGLT2i = sodium-glucose cotransporter 2 inhibitor. | |

| Supplemental Table 3. Crude Analysis of 30-Day All-Cause Readmission | | |
| --- | --- | --- |
| Group | **HR (95% CI)** | **P Value** |
| Monotherapy | Reference | |
| Dual therapy | 0.84 (0.69-1.02) | 0.072 |
| Triple therapy | 0.81 (0.65-1.01) | 0.064 |
| Quad therapy | 0.64 (0.48-0.86) | 0.003 |
| Hazard ratios (HR) and 95% confidence intervals (CI) are shown. | | |

| Supplemental Table 4. Crude Analysis of 30-Day All-Cause Death or All-Cause Readmission | | |
| --- | --- | --- |
| Group | **HR (95% CI)** | **P Value** |
| Monotherapy | Reference | |
| Dual therapy | 0.83 (0.68-1.00) | 0.048 |
| Triple therapy | 0.77 (0.62-0.95) | 0.017 |
| Quad therapy | 0.62 (0.47-0.83) | 0.001 |
| Hazard ratios (HR) and 95% confidence intervals (CI) are shown. | | |

| Supplemental Table 5. Crude Analysis of 30-Day Heart Failure Readmission | | |
| --- | --- | --- |
| Group | **HR (95% CI)** | **P Value** |
| Monotherapy | Reference | |
| Dual therapy | 0.85 (0.67-1.07) | 0.165 |
| Triple therapy | 0.88 (0.68-1.13) | 0.313 |
| Quad therapy | 0.65 (0.46-0.92) | 0.015 |
| Hazard ratios (HR) and 95% confidence intervals (CI) are shown. | | |

| Supplemental Table 6. Crude Analysis of 30-Day All-Cause Death or Heart Failure Readmission | | |
| --- | --- | --- |
| Group | **HR (95% CI)** | **P Value** |
| Monotherapy | Reference | |
| Dual therapy | 0.85 (0.68-1.06) | 0.146 |
| Triple therapy | 0.81 (0.63-1.03) | 0.090 |
| Quad therapy | 0.62 (0.44-0.86) | 0.005 |
| Hazard ratios (HR) and 95% confidence intervals (CI) are shown. | | |

**
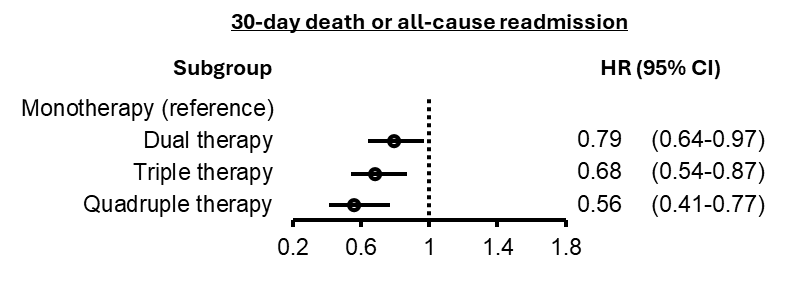
**

**Supplemental Figure 1. Forest Plot for a Composite Endpoint of 30-Day All-Cause Readmission or All-Cause Death.** Hazard ratios (HR) and 95% confidence intervals (CI) are shown.

**
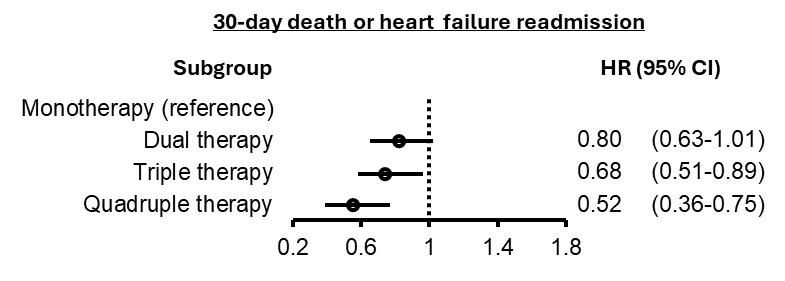
**

**Supplemental Figure 2. Forest Plot for a Composite Endpoint of 30-Dat Heart Failure Readmission or All -Cause Death.** Hazard ratios (HR) and 95% confidence intervals (CI) are shown.
